# General population’s knowledge about the anatomical locations of organs and medical terms today and 50 years ago: a replication study

**DOI:** 10.3205/zma001490

**Published:** 2021-06-15

**Authors:** Sigrid Harendza, Anne Münter, Lisa Bußenius, Anja Bittner

**Affiliations:** 1 University Medical Center Hamburg-Eppendorf, III. Department of Internal Medicine, Hamburg, Germany; 2 University Medical Center Hamburg-Eppendorf, Center for Experimental Medicine, Department of Biochemistry and Molecular Cell Biology, Hamburg, Germany; 3 Bielefeld Medical School OWL, Deanery, Bielefeld, Germany

**Keywords:** anatomy, communication, cross-sectional study, health literacy, medical knowledge, medical terms

## Abstract

**Background: **Physicians are frequently not aware that patients may not be familiar with the meaning of medical terms or have limited knowledge about the location of organs. These aspects of functional health competence require particular attention when designing communication curricula for undergraduate medical students. The aim of our study was to evaluate the knowledge of laypersons about the anatomical locations of organs and the definitions of commonly used medical terms as relevant aspects of health literacy. Furthermore, we wished to compare it with the knowledge of a historical patient cohort who performed this study 50 years ago.

**Methods:** In this replication study, multiple-choice questionnaires with simple anatomy and common medical terms which were published in 1970 were distributed among a convenience sample of lay volunteers (n=537) from the streets of Hamburg, Germany. Sociodemographic data including sex, age, highest educational school achievement, occupation in a field associated with medicine, and German as first language were also collected. The percentage of laypersons’ correct answers was compared to the percentage of correct answers of a historical patient cohort (n=234) published in 1970 to identify the development of health literacy as basis for curricular planning.

**Results: **Laypersons showed significantly more correct answers in four of eight simple anatomical locations of organs (p<0.001). For seven commonly used medical terms laypersons only gave significantly more correct answers for the definitions of “jaundice” (p<0.001) and “diarrhoea” (p=0.001) compared to the historical cohort from 1970. Participants with a senior high school degree performed significantly better with respect to total scores of correct organ locations (*p*<0.001, *d*=0.35) and correct definitions of medical terms (*p*=0.001, *d*=0.30) than participants who completed junior high school.

**Conclusion: **The definitions of common medical terms and the correct anatomical locations of organs by laypersons have increased during the past 50 years but could still need improvement by school education and media information of better quality. Medical educators should know about the low health literacy of laypersons with respect to these aspects to raise medical students’ awareness for this problem and to provide communication training for medical students to use comprehensible language during history taking and shared decision making.

## Zusammenfassung

**Hintergrund: **Ärzt*innen ist häufig nicht bewusst, dass Patient*innen möglicherweise nicht mit der Bedeutung medizinischer Begriffe vertraut sind oder nur begrenzte Kenntnisse über die Lage von Organen haben. Diese Aspekte der funktionalen Gesundheitskompetenz erfordern besondere Aufmerksamkeit bei der Gestaltung von Kommunikationscurricula für Medizinstudierende. Ziel unserer Studie war es, das Wissen von medizinischen Laien über die anatomische Lage von Organen und die Definitionen gebräuchlicher medizinischer Begriffe als relevante Aspekte der Gesundheitskompetenz zu evaluieren. Außerdem wollten wir es mit dem Wissen einer historischen Patient*innenkohorte vergleichen, die vor 50 Jahren an dieser Studie teilgenommen hat.

**Methoden: **In dieser Replikationsstudie wurden Multiple-Choice-Fragebögen mit einfacher Anatomie und gebräuchlichen medizinischen Begriffen, die 1970 veröffentlicht wurden, an eine Zufallsstichprobe von freiwilligen medizinischen Laien (n=537) auf den Straßen Hamburgs, Deutschland, verteilt. Soziodemographische Daten wie Geschlecht, Alter, höchster Schulabschluss, berufliche Tätigkeit in einem mit der Medizin assoziierten Bereich und Deutsch als Erstsprache wurden ebenfalls erhoben. Der Prozentsatz der richtigen Antworten der medizinischen Laien wurde mit dem Prozentsatz der richtigen Antworten einer historischen Patient*innenkohorte (n=234) aus dem Jahr 1970 verglichen, um die Entwicklung der Gesundheitskompetenz als Grundlage für die Curriculumsplanung zu ermitteln.

**Ergebnisse: **Medizinische Laien zeigten bei vier von acht einfachen anatomischen Lagen von Organen signifikant mehr richtige Antworten (p<0,001). Bei sieben häufig verwendeten medizinischen Begriffen gaben medizinische Laien nur bei den Definitionen von „Gelbsucht“ (p<0,001) und „Durchfall“ (p=0,001) signifikant mehr richtige Antworten im Vergleich zur historischen Patient*innenkohorte von 1970. Teilnehmende mit einem höheren Schulabschluss schnitten in Bezug auf die Gesamtpunktzahl der korrekten Organlokalisationen (p<0,001, d=0,35) und der korrekten Definitionen von medizinischen Begriffen (p=0,001, d=0,30) signifikant besser ab als Teilnehmende mit einem mittleren Schulabschluss.

**Schlussfolgerung: **Die Definitionen gängiger medizinischer Begriffe und die korrekte Bestimmung der anatomischen Lage von Organen durch medizinische Laien haben in den letzten 50 Jahren zugenommen, könnten aber immer noch durch Schulbildung und qualitativ bessere Medieninformationen verbessert werden. Medizinische Ausbilder*innen sollten über die geringe Gesundheitskompetenz von medizinischen Laien in Bezug auf diese Aspekte Bescheid wissen, um Medizinstudierende für dieses Problem zu sensibilisieren und ein Kommunikationstraining für Medizinstudierende anzubieten, damit diese bei der Anamneseerhebung und gemeinsamen Entscheidungsfindung eine verständliche Sprache verwenden.

## 1. Introduction

Health literacy, the ability to read, understand and process basic health information, is needed to interact with health care providers and to make appropriate health decisions [1]. Lower health literacy is associated with poorer health care outcomes and reduced use of health care services [2]. A UK-based study found in 2015, that 61% of the participants were below the text and numeracy threshold to understand and use commonly used English health information materials [3]. In 2017, 54.3% of the participants in a cross-sectional study in Germany were identified to have limited health literacy [4].

Physicians are frequently not aware that patients may not be familiar with the meaning of medical terms. Residents used technical medical terms in 66.2% of the interactions with patients during history taking [5]. Residents reported to use plain language in 88%, but used two jargon medical terms per minute in a low health literacy standardized patient encounter [6]. A health literacy communication training program for residents increased their use of plain language from 33% to 86% [7]. Medical students who worked with individuals with low health literacy showed more knowledge and skills confidence regarding health literacy [8].

In 1970, Boyle published a study under the heading “contemporary themes” in the British Medical Journal about 234 laypersons’ understanding of commonly used medical terms and knowledge of anatomical locations of organs, which turned out to be low [9]. In the meantime, school education has improved and includes more health aspects [10] and a lot of health information is available via new media [11], [12], [13]. However, a cross-sectional study with inpatients of a German hospital who were asked to define medical terms in 2019 revealed that more patients claimed to know the meaning of medical terms than it was the case in the actual test [14]. Furthermore, the number of correct answers in the test did not correlate with reading the newspaper or watching TV.

We were wondering how laypersons would perform answering Boyle’s questionnaires [9] almost 50 years later. The purpose of this study was to describe laypersons’ medical knowledge with respect to their sociodemographic background as a starting point to raise health care personnel’s and medical students’ awareness when communicating with patients. We also wished to explore whether differences in the correct answers regarding the anatomical locations of different organs and the definitions of common medical terms could be observed between today’s laypersons and the historical patient cohort. 

## 2. Methods

### 2.1. Design of the questionnaires

**Figure 1:**
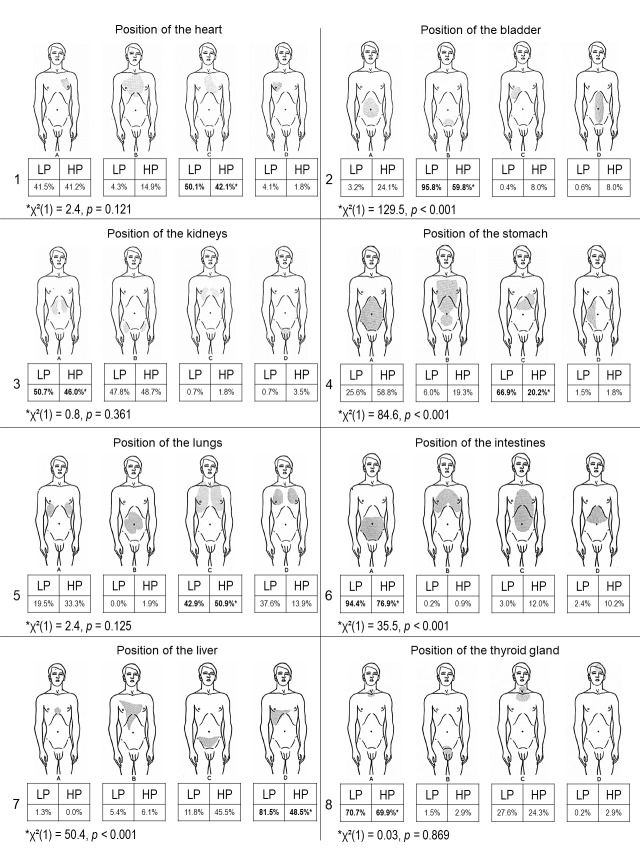
Anatomical localizations of organs [9]. LP: laypersons, HP: historical patient cohort. 1: “Heart” (LP: n=533, HP: n=114), 2: “Bladder” (LP: n=534, HP: n=112), 3: “Kidneys” (LP: n=534, HP: n=113), 4: “Stomach” (LP: n=532, HP: n=114), 5: “Lungs” (LP: n=534, HP: n=108), 6: “Intestines” (LP: n=536, HP: n=108), 7: “Liver” (LP: n=535, HP: n=99), 8: “Thyroid gland” (LP: n=536, HP: n=103). **: p<0.001.

Boyle designed two multiple choice questionnaires, one with pictures of anatomical organ locations and the other with definitions of some common medical terms [9]. We used the original anatomical drawings with four possible answers for the organ location of “heart”, “bladder”, “kidneys”, “stomach”, “lungs”, “intestines”, “liver”, and “thyroid gland” (see figure 1 (Fig. 1)). A maximum total score of eight could be reached for eight correct answers. Of the 12 medical terms with five possible definitions we included the seven terms “arthritis”, “heartburn”, “jaundice”, “diarrhoea”, “constipation”, “bronchitis”, and “piles”. The terms “least starchy food”, “a medicine”, “palpitation”, “a good appetite”, and “flatulence” were omitted, because they are not regularly used anymore. A maximum total score of seven could be reached for seven correct answers. The questionnaires were translated into German. All questions had only one correct answer. Participants were asked to provide the following sociodemographic data: sex, age, their highest educational school achievement, whether they worked in a field associated with medicine and whether German was their first language.

#### 2.2. Study design and participants

In summer and autumn 2017, 537 laypersons, at least 18 years old, were randomly approached in the streets of different neighbourhoods of Hamburg to collect a convenience sample and asked to participate in this study. When oral informed consent for voluntary participation was given, the participants received the paper-based questionnaires and a pen like the historical patient cohort [9] and were asked to answer all questions while they had no access to information to find the correct answers. The historical patient cohort from 1970 included a convenience sample of 234 patients, 17 years and older, who attended an outpatient clinic at the Southern General Hospital in Glasgow for the first time [9]. Sociodemographic data of all participants are given in table 1 (Tab. 1). This study was performed according to the standards of the Declarations of Helsinki and Geneva. It was exempt from ethical approval by the Ethics Committee of the Chamber of Physicians, Hamburg, because no experiments were done with participants. All participants gave oral consent and their data were anonymized.

#### 2.3. Data analysis

Statistical analyses were performed with IBM SPSS Statistics 26.0 (Armonk, NY: IBM Corp.). Chi-square tests were calculated to compare the answers of laypersons and the historical patient cohort. The level of significance for all findings was set to p<0.05. To identify significant differences between our cohort and the historical cohort with a mean effect of w=.3 and a power of 95%, a sample size of n=145 participants was needed and reached by our sampling. To identify a possible influence of sociodemographic factors on medical knowledge we compared the respective total scores of correct anatomical locations of organs and definitions of medical terms with independent t tests and estimated the effect size with Cohen’s d in our cohort of laypersons. 

## 3. Results

A total of 537 questionnaires were received from laypersons and 234 patients participated in the historical cohort [9]. The sociodemographic data are displayed in table 1 (Tab. 1). On average, the laypersons in our cohort were 42.0±19.3 years old and the individuals from the historical patient cohort [9] were 43.2 years old. Of the laypersons, 63.0% were female, 36.3% were male, and 0.7% were diverse. The sex distribution of the historical patient cohort [9] was 66.7% female and 33.3% male. 

With respect to the anatomical localizations of organs laypersons gave significantly (p<0.001) more correct answers for the anatomical localization of the “bladder”, the “stomach”, the “intestines”, and the “liver” compared to the individuals form the historical patient cohort (see figure 1 (Fig. 1)). The results of the laypersons’ and the historical patient cohort’s answers defining common medical terms are shown in attachment 1 . Of the seven definitions, laypersons gave significantly more correct answers (see attachment 1 , correct answers marked in bold) only for the definitions of “jaundice” (p<0.001) and “diarrhoea” (p=0.001). We found significant small to medium differences with respect to sociodemographic factors for both total scores (see table 2 (Tab. 2)). With small effect sizes (d=0.29 for organ localisation and d=0.25 for medical terms), female participants performed significantly better than male participants and participants with a senior high school degree performed significantly better with medium effect sizes (d=0.35 for organ localisation and d=0.35 for medical terms) than participants who completed junior high school. Participants working in a field associated with medicine had significantly higher total scores with medium effect sizes (d=0.78) than participants from other fields in both total scores. Participants speaking German as first language performed significantly better on the definitions of medical terms with a small effect (d=0.46) but showed no significant differences regarding the anatomical localizations of organs.

## 4. Discussion

In 1970, health literacy with respect to the anatomical locations of organs and common medical terms was fairly low [9]. In our study, 50 years later, we found that the percentage of correct definitions had improved significantly in laypersons in only 28.6% and in correctly defining the location of organs in 50%. Additionally, laypersons with a senior high school degree scored significantly better than laypersons with a junior high school degree. These findings could underscore, that health education in school could to be improved to empower laypersons to understand their bodies better to lead a healthy life [15]. However, we do not know whether participants in our study acquired their medical knowledge at school or by other means. While general health education at school had little effect, a specific teaching program led to better health literacy in children [16]. Furthermore, the availability of health information in the media could also have led to some improvement in the questionnaire by our cohort of laypersons compared to the historical cohort. However, presentation of health issues in the media can oversimplify health issues and thereby distort health information [17]. This could be a hint that it is difficult for laypersons to identify reliable sources of health information in the media. Especially in the internet, many sources of health information were found to be of suboptimal quality [18]. While anatomical knowledge in a study was generally poor, participants in health-related employment scored significantly higher [19]. We could confirm this finding in our cohort. Medical students and physicians should be aware of the difficulties laypersons without a professional health-related background still have today in knowing about the correct locations of organs and about the definitions of common medical terms when communicating with patients and learn techniques to avoid misunderstandings.

Even though we found significant differences between male and female participants with respect to organ localizations and medical terms, effect sizes were low and it remains unknown whether these differences are relevant for making competent health decisions. However, medical terms are often colloquially used but not fully understood by laypersons. They were developed for physicians to communicate with each other [20] and are usually acquired during undergraduate medical training [21], [22]. On the other hand, medical students who voluntarily translate medical documents into plain language use plain language more frequently in simulated physician-patient encounters [23]. This is one way of raising awareness in future physicians for the inherent dangers of misunderstandings in physician-patient communication when using presumably “common” medical terms. When an attitude of feeling powerful when using medical jargon while talking to patients [24] persists, it can lead to potentially dangerous miscommunication between physicians and patients. This problem can be overcome by raising awareness for the different ways to communicate as a physician [25], which found entry in a European consensus on communication in health professions [26].

Our study has some limitations. Since it was not performed in the same country as the original study, the difference in school and health care systems might have had an effect on the results. The participating laypersons were not completely randomly selected but randomly approached and volunteered to participate which might have led to a selection of very motivated or health literate participants. Even though participants were approached in different neighbourhoods of the city of Hamburg, our convenience sample cannot be representative for the German population. Furthermore, the Hamburg cohort and the historical cohort consist of >60% female participants, which is not representative for the general population. An additional sampling bias of the Hamburg cohort is the high percentage of participants who attended senior high school. The original questionnaire could only be used for the locations of the organs but not for all medical terms, because some of these terms are not colloquially used anymore. Overall, the questionnaire contains a very limited number of questions due to the historical template and the necessary reduction of items.

Despite these limitations, our study shows that after 50 years of working on better health communication laypersons still have difficulties to name the location of organs correctly or to define medical terms, even though there has been improvement to a certain extent. It cannot be assumed that better access to new media automatically increases health literacy. Medical students need to learn during their undergraduate training that health literacy in laypersons cannot be taken for granted. They need the support of medical educators in their respective medical curricula to develop strategies to communicate with patients comprehensibly as a basis for shared decision making.

## 5. Conclusion

Despite the growing amount of health information available to the general public medical students, physicians, and health personnel need to be aware that deficits in knowledge about organ locations and medical terms still exist in laypersons. Being aware of these deficits can be a first step towards preventing misunderstandings when communicating with patients. Education at school seems to be a good way to apply some leverage as well as good quality health information in the media. Medical students need to become aware that the use of plain language to explain complex medical contexts is necessary in physician-patient communication for patient-centred healthcare and that health literacy cannot be taken for granted. Communication curricula should provide medical students with the opportunity to exercise this particular communication skill. 

## Acknowledgements

We would like to thank all participants who devoted their time and effort to this study.

## Competing interests

The authors declare that they have no competing interests. 

## Supplementary Material

Distribution of definitions of common medical terms

## Figures and Tables

**Table 1:**
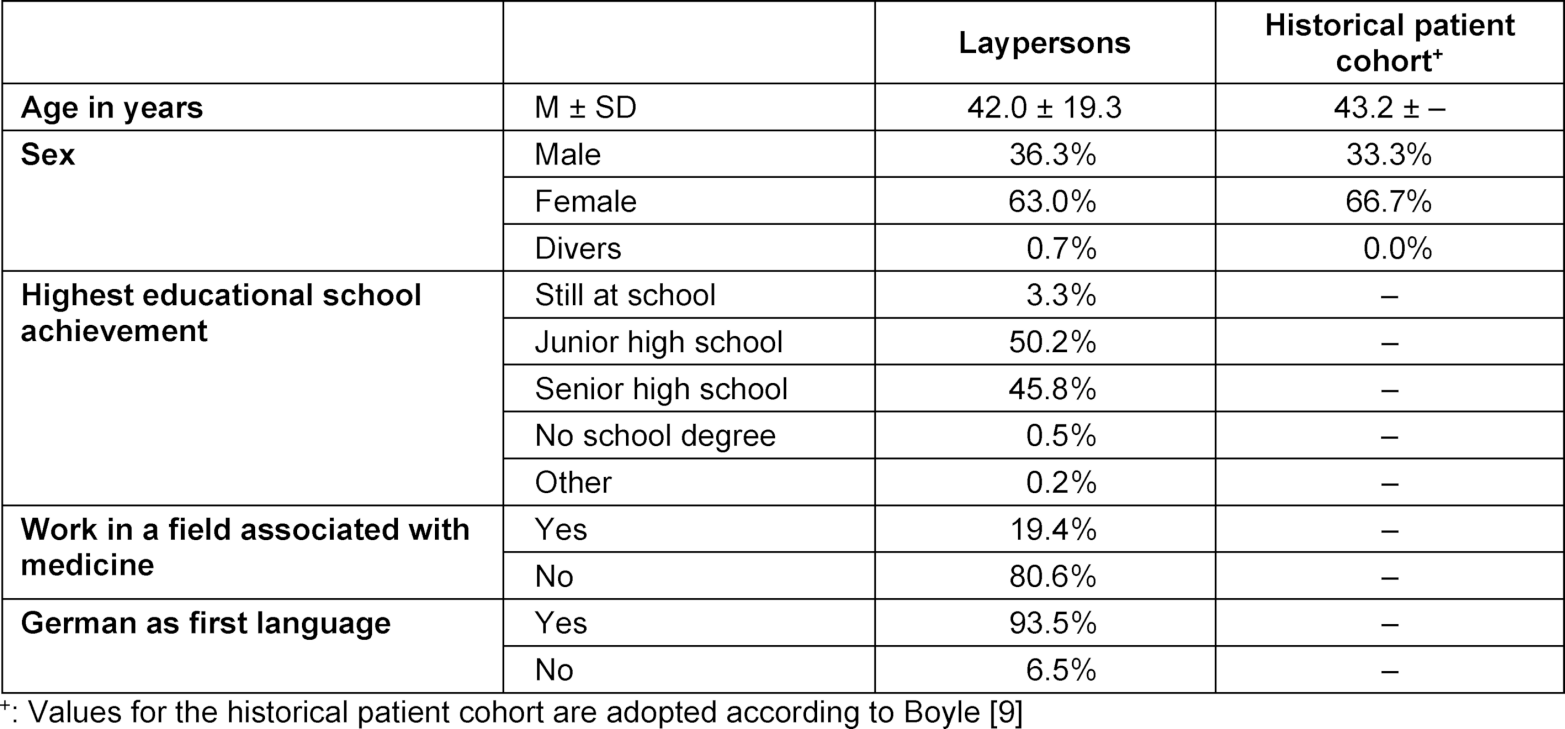
Sociodemographic data of laypersons and the historical patient cohort

**Table 2:**
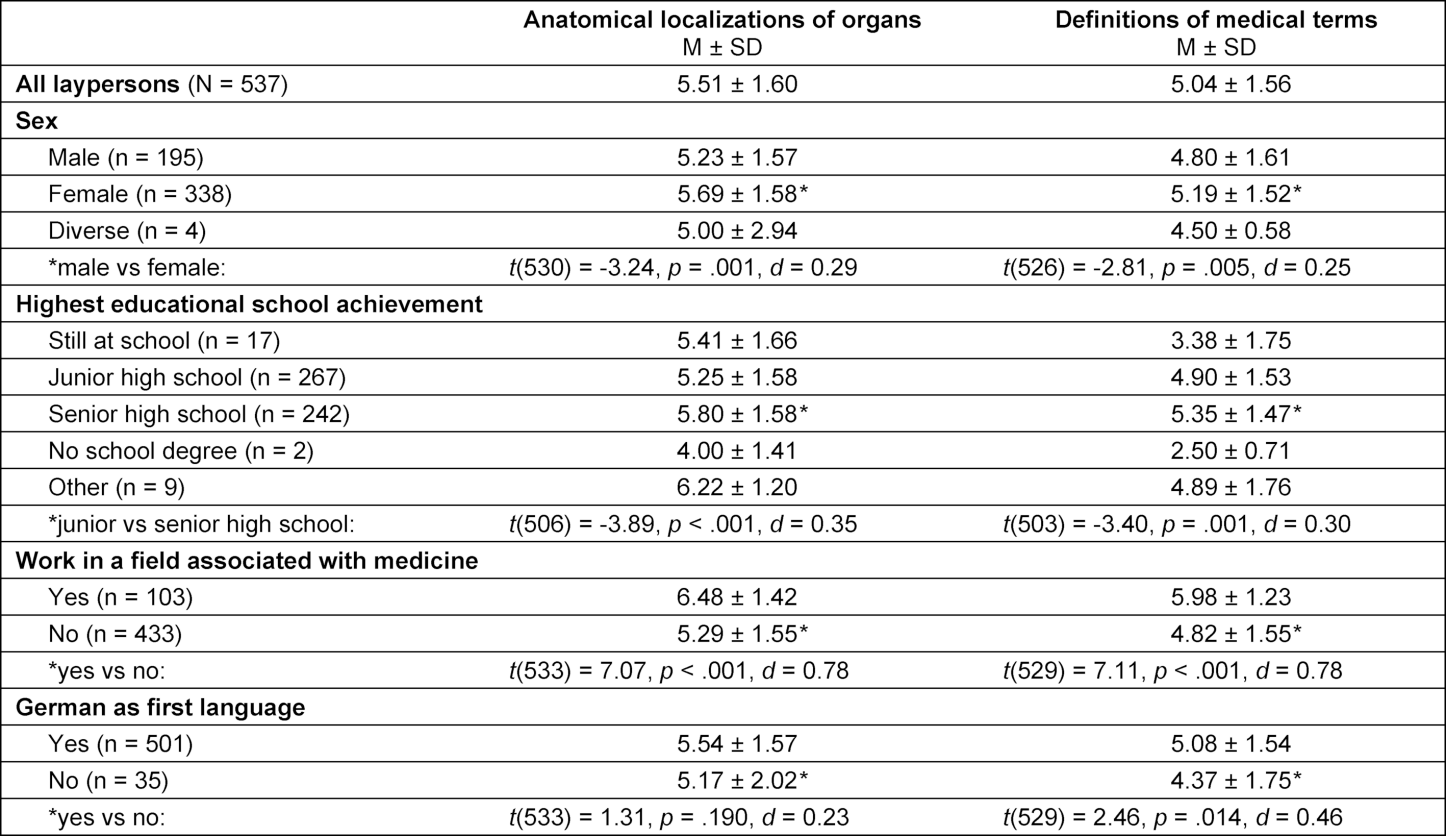
Total scores for anatomical localization of organ positions and definitions of medical terms
