# Supplementary material for: General population’s knowledge about the anatomical locations of organs and medical terms today and 50 years ago: a replication study
Source: GMS J Med Educ. 2021 Jun 15;38(5):Doc94. doi: 10.3205/zma001490 (PMC8256121; doi:10.3205/zma001490)
Supplement: Distribution of definitions of common medical terms [file JME-38-5-94-s-001.pdf]

Attachment 1: Distribution of definitions of common medical terms

1. Definition of „Arthritis“

Definitions	Laypersons n = 518	Historical patient cohort n = 113
A disease due to muscle strain or a “pulled muscle”.	32 (6.2%)	5 (4.4%)
Any disease causing a painful back.	9 (1.7%)	1 (0.9%)
Any disease with sore arms or legs.	24 (4.6%)	9 (8.0%)
Lumbago.	8 (1.5%)	1 (0.9%)
Any disease causing a painful joint or painful joints.	**445 (85.9%)**	**97 (85.8%)***
	*$\chi^2(1) = 0$, $p = .985$	

2. Definition of „Heartburn“

Definitions	Laypersons n = 532	Historical patient cohort n = 112
Passage of wind through the mouth.	27 (5.0%)	9 (8.0%)
Excess saliva.	59 (11.1%)	5 (4.5%)
A burning sensation behind the breastbone.	**422 (79.3%)**	**95 (84.8%)***
A dull ache in the stomach.	14 (2.7%)	1 (0.9%)
A feeling of the heart thumping inside the chest.	10 (1.9%)	2 (1.8%)
	*$\chi^2(1) = 1.77$, $p = .184$	

3. Definition of „Jaundice“

Definitions	Laypersons n = 525	Historical patient cohort n = 111
Generally sick and unwell – with yellow vomit.	8 (1.5%)	11 (9.9%)
Pale skin and anaemia (bloodlessness).	21 (4.0%)	1 (0.9%)
Generally unwell, whether yellow skin or not.	8 (1.5%)	6 (5.4%)
Yellow skin and eyes.	**473 (90.1%)**	**85 (76.6%)***
Headache, furred yellowish tongue, and “run down” feeling.	15 (2.9%)	8 (7.2%)
	*$\chi^2(1) = 15.56$, $p < .001$	

4. Definition of „Diarrhoea“

Definitions	Laypersons n = 505	Historical patient cohort n = 108
Passing loose bowel motions.	**277 (54.9%)**	**40 (37.0%)***
Opening one’s bowels more than once a day.	24 (4.8%)	4 (3.7%)
Passing a lot of bowel motions in a short time.	142 (28.1%)	59 (54.6%)
Straining to pass bowel motions.	36 (7.1%)	4 (3.7%)
Passing a lot of wind by the back passage.	26 (5.1%)	1 (0.9%)
	*$\chi^2(1) = 11.31$, $p < .001$	

5. Definition of „Constipation“

Definitions	Laypersons n = 494	Historical patient cohort n = 107
Passing a lot of wind by the back passage.	91 (18.4%)	6 (5.6%)
Passing loose bowel motions.	44 (8.9%)	0 (0.0%)
Passing dark-coloured motions.	49 (9.9%)	1 (0.9%)
Difficulty in opening one’s bowels.	**264 (53.4%)**	**64 (59.8%)***
Not opening one’s bowels every day.	46 (9.3%)	36 (33.6%)
	*$\chi^2(1) = 1.44$, $p < .230$	

6. Definition of „Bronchitis“

Definitions	Laypersons n = 521	Historical patient cohort n = 106
Any disease causing a bad cough.	65 (12.5%)	3 (2.8%)
Wheezing, cough, and spit; usually worse in winter.	**392 (75.2%)**	**85 (80.2)%***
Severe influenza.	29 (5.6%)	3 (2.8%)
Breathlessness.	18 (3.5%)	9 (8.5%)
Any chest complaint.	17 (3.3%)	6 (5.7%)
	*$\chi^2(1) = 1.19$, $p = .276$	

Attachment 1 to: Harendza S, Münter A, Bußenius L, Bittner A. *General population's knowledge about the anatomical locations of organs and medical terms today and 50 years ago: a replication study.* GMS J Med Educ. 2021;38(5):Doc94. DOI: 10.3205/zma001490

**7. Definition of „Piles“**		
**Definitions**	**Laypersons** n = 525	**Historical patient cohort** n = 102
Any disease of back passage, whether painful or not.	36 (6.9%)	1 (1.0%)
Any disease of back passage, but usually painful.	11 (2.0%)	3 (2.9%)
Enlarged blood vessels at the pack passage.	**410 (78.1%)**	**75 (73.5%)***
Any pain at the back passage.	45 (8.6%)	6 (5.9%)
Any bleeding at the back passage.	23 (4.4%)	17 (16.7%)
	*$\chi^2(1) = 1.02$, $p = .313$	
